# COMP report: CPQR technical quality control guidelines for radiation treatment centers

**DOI:** 10.1002/acm2.12295

**Published:** 2018-02-26

**Authors:** Kyle E. Malkoske, Michelle K. Nielsen, Laurent Tantôt, Natalie Pomerleau‐Dalcourt, Marie‐Pierre Milette, Kevin R. Diamond, Normand Frenière, Marie‐Joëlle Bertrand, J. Eduardo Villarreal‐Barajas, David K. Sasaki, Jason Schella, John Grant, L. John Schreiner, Jean‐Pierre Bissonnette

**Affiliations:** ^1^ Radiation Treatment Program Simcoe Muskoka Regional Cancer Program Royal Victoria Regional Health Centre Barrie ON Canada; ^2^ Department of Medical Physics Mississauga Halton/Central West Regional Cancer Program Trillium Health Partners Mississauga ON Canada; ^3^ Département de Radio‐Oncologie Hôpital Maisonneuve‐Rosemont Montréal QC Canada; ^4^ Département de Physique Médicale Centre d'oncologie Dr. Léon‐Richard Réseau de Santé Vitalité Moncton NB Canada; ^5^ Department of Medical Physics British Columbia Cancer Agency – Centre for the Southern Interior Kelowna BC Canada; ^6^ School of Interdisciplinary Science McMaster University Hamilton ON Canada; ^7^ Department of Medical Physics Juravinski Cancer Centre Hamilton ON Canada; ^8^ Département de Radio‐Oncologie Centre intégré universitaire de santé et de services sociaux de la Mauricie‐et‐du‐Centre‐du‐Québec Centre hospitalier affilié universitaire régional Trois‐Rivières QC Canada; ^9^ Département de Radio‐Oncologie Centre de santé et de services sociaux de Chicoutimi Chicoutimi QC Canada; ^10^ Department of Oncology University of Calgary Calgary AB Canada; ^11^ Department of Medical Physics Tom Baker Cancer Centre Calgary AB Canada; ^12^ Division of Medical Physics CancerCare Manitoba Winnipeg MB Canada; ^13^ Department of Radiation Oncology Dalhousie University Halifax NS Canada; ^14^ Medical Physics Team QEII Health Sciences Centre Nova Scotia Health Authority Halifax NS Canada; ^15^ Cape Breton Cancer Centre Nova Scotia Health Authority Sydney NS Canada; ^16^ Departments of Oncology and Physics Queen's University Kingston ON Canada; ^17^ Department of Medical Physics Cancer Centre of Southeastern Ontario at KGH Kingston ON Canada; ^18^ Department of Radiation Oncology University of Toronto Toronto ON Canada; ^19^ Department of Medical Physics Princess Margaret Cancer Centre Toronto ON Canada

**Keywords:** Canada, guidelines, quality assurance, radiation safety, technical quality control

## Abstract

The Canadian Organization of Medical Physicists (COMP), in close partnership with the Canadian Partnership for Quality Radiotherapy (CPQR) has developed a series of Technical Quality Control (TQC) guidelines for radiation treatment equipment. These guidelines outline the performance objectives that equipment should meet in order to ensure an acceptable level of radiation treatment quality. The TQC guidelines have been rigorously reviewed and field tested in a variety of Canadian radiation treatment facilities. The development process enables rapid review and update to keep the guidelines current with changes in technology. This announcement provides an introduction to the guidelines, describing their scope and how they should be interpreted. Details of recommended tests can be found in separate, equipment specific TQC guidelines published in the JACMP (COMP Reports), or the website of the Canadian Partnership for Quality Radiotherapy (www.cpqr.ca).

## 1 INTRODUCTION

The Canadian Organization of Medical Physicists (COMP) has led the development of technical quality control (TQC) guidelines for radiation treatment systems. This work was facilitated by the Canadian Partnership for Quality Radiotherapy (CPQR), an alliance amongst the three key national professional organizations involved in the delivery of radiation treatment in Canada (COMP, the Canadian Association of Radiation Oncology (CARO), and the Canadian Association of Medical Radiation Technologists (CAMRT)). Financial and strategic backing is provided by the federal government through the Canadian Partnership Against Cancer (CPAC), a national resource for advancing cancer prevention and treatment. The vision and mandate of the CPQR is to support the universal availability of high quality and safe radiotherapy for all Canadians through initiatives aimed at improving quality and mitigating risk.

In a recent publication, we introduced the rationale, development process, and impact of the TQC guidelines in Canada.^1^ This announcement serves as a preface to the guidelines themselves, and provides details on how the performance objectives and criteria in the guidelines should be interpreted. The TQC guidelines supersede an earlier set of equipment quality control documents that were produced under the sponsorship of the Canadian Association of Provincial Cancer Agencies (CAPCA).^2^

Ownership of the CPQR documents resides jointly with the national professional organizations involved in the delivery of radiation treatment in Canada — COMP, CARO, CAMRT, and CPAC. Decisions regarding content changes to the TQC guidelines reside with COMP and are made in close partnership with the CPQR Steering Committee and partners.

## 2 SCOPE

The suite of TQC guidelines outline the minimum performance objectives and safety criteria that equipment or technology should meet in order to assure safe operation and an acceptable level of performance. The development of the individual TQC guidelines is spearheaded by expert reviewers and involves broad stakeholder input from the medical physics and radiation oncology community, including field testing.^1^ Guidelines are reviewed and updated every 5 yr, or earlier if necessary due to changes in technology or application, with the most current versions available on the CPQR website in both English^3^ and French.^4^ It is the responsibility of the supervising physicist to ensure that locally available test equipment and procedures are sufficiently sensitive to establish compliance with the criteria specified within the suite of TQC guidelines.

All information contained in the TQC guidelines is intended to be used at the discretion of each individual center to help guide quality and safety program improvement. There are no legal standards supporting the guidelines; specific federal or provincial regulations and license conditions take precedence over the content of the guidelines. That said, adherence to the TQC guidelines is a nationally recognized quality practice, as these guidelines are specifically referenced in Canada's national hospital accreditation process (Accreditation Canada's Qmentum module for Cancer Care programs).^5^

The detailed performance objectives and safety criteria for radiation treatment equipment are itemized in a series of equipment specific TQC guidelines that can be accessed on the CPQR website^3^, ^4^ and in the COMP Reports section of this journal. These guidelines have independent generation dates and review cycles. Each equipment‐specific TQC guideline contains a brief system description and the tables of recommended tests, frequencies, and performance objectives. Radiation safety activities, such as those outlined by regulators^6^ and the CPQR's *Quality Assurance Guidelines for Canadian Radiation Treatment Programs* 7 shall be integrated into routine quality control for equipment. The TQC guidelines include testing of the facility's radiation safety systems in a separate guideline to promote a consistent approach to safety for facilities and regulators.^8^ Comprehensive quality assurance programs shall adhere to the TQC guidelines of both the equipment and its associated safety systems.

## 3 PERFORMANCE OBJECTIVES AND CRITERIA

Objectives and criteria used in the performance evaluation of radiation treatment equipment and technologies fall into several categories:

- Functionality — Equipment systems and subsystems for which the criteria of performance are “functional” are either working correctly or not. Such systems are commonly associated with the safety features of the equipment or installation.
- Reproducibility — The results of routine quality control tests, for which reproducibility is the criterion, are assessed against the baseline results obtained from the unit during acceptance testing and/or commissioning. Tolerances and action levels should be set for parameters that can be quantified.
- Accuracy — Quality control tests which measure accuracy are designed to assess the deviance of a measured parameter from its expected or defined value. An example would be a test quantifying positional accuracy.
- Characterization and documentation — In some cases it is necessary to take measurements to characterize the performance of a piece of equipment before it can be used clinically. An example is the measurement of the ion collection efficiency of an ionization chamber.
- Completeness — The use of this term is restricted to the periodic review of quality control procedures, analysis, and documentation.

For quantities that can be measured, tolerance and action levels are defined as follows.

- Tolerance level — The tolerance level is used to describe the normal operating range of a system performance parameter. If the difference between the measured value and its expected or defined value is at, or within, the stated tolerance level then no further action is required. The tolerance level will be impacted by the intrinsic variation in the system, as well as the precision of the equipment and process used to measure the given parameter. Statistical methods for analyzing quality control data may be applied to set appropriate tolerance levels ( e.g., the control limits used in statistical process control [SPC] charts).^9^, ^10^ The TQC guidelines provide recommendations for tolerance levels, based on typical equipment and experience, which may be adapted due to local observations. However, equipment and processes should be selected such that the tolerance levels are less than, or well within, the action levels defined below.
- Action level — The action level corresponds to a clinically relevant specification. That is, when the performance parameter exceeds its action level, the deviation may pose a clinically significant impact. If the difference between the measured value and its expected or defined value exceeds the action level, then an investigation is required immediately, and the equipment is taken out of clinical use. The investigation should identify whether the deviation is random or systematic through repeat measurement, ideally with independent equipment and/or personnel. The ideal response is to bring the system back to a state of functioning that meets all tolerance levels. If this is not immediately possible, then the use of the equipment shall be restricted to clinical situations in which the identified deviation is of no, or acceptable, clinical significance. Any restrictions on clinical operations must be clearly communicated to the users of the equipment and others as appropriate. Furthermore, the use of the restricted operation must be inhibited by means of hardware locks and/or software administration settings, if possible, in order to prevent inadvertent use.

If the difference between the measurement and its expected or specified value lies between the tolerance and action levels, several courses of action are open. A decision may be made to monitor the performance of the parameter in question over a period of time and postpone a decision until the behavior of the parameter is appropriately characterized. For a problem that is easily and quickly rectifiable, remedial action may be taken as soon as possible. Alternatively, remedial action may be delayed to limit impact on clinical workflow. These options and actions should be described clearly in the facility's quality control policies and procedures.

The decision as to which course of action is most appropriate when tolerance or action levels are exceeded should be made by a qualified medical physicist (QMP) in consultation with other clinical and administrative staff in the program. An appropriate subset of acceptance, commissioning, or routine quality control tests shall be performed after any repair of the equipment. The extent of testing required shall be judged by a QMP.

## 4 QUALITY CONTROL OF EQUIPMENT

The purpose of quality control testing is to assure that operational standards that were considered acceptable at time of purchase continue to be maintained, as closely as possible, over the life of the equipment.^11^ Thus, quality‐control testing involves periodic repetitions, partial or full, of acceptance and commissioning tests. Tests shall be performed by a QMP, or a suitably trained individual working under the supervision of a QMP. A second QMP shall independently verify the implementation, analysis, and interpretation of the quality control tests at least annually. This independent check shall be documented.

Ideally, daily tests shall be scheduled prior to patient treatments. Testing at less than the frequency recommended in the TQC guidelines is acceptable only if data‐driven experience has established that the parameters of interest are highly stable. Documented evidence supporting this decision is required.

Preventive maintenance schedules and interventions recommended by the manufacturer of the equipment shall be adhered to. Frequently, equipment repairs and quality control testing are performed by different individuals. Good communication and reporting between the various staff involved are essential.

Appropriate documentation is a required component of a quality assurance program. Documentation should be separated into two major categories: protocols and records. The quality control protocol should provide sufficient detail concerning the test equipment and procedures to be followed so that there is no ambiguity in the interpretation of the test results. They should also clearly define actions to be taken if tests fall outside of action levels. The quality control record contains the results of the tests, the date(s) on which they were performed, and the name of the tester and the supervising QMP, as appropriate.

## CONFLICT OF INTEREST

The authors have no conflicts of interest to declare.
